# Mechanism of regulating macrophages/osteoclasts in attenuating wear particle-induced aseptic osteolysis

**DOI:** 10.3389/fimmu.2023.1274679

**Published:** 2023-10-04

**Authors:** Zhaoyang Yin, Ge Gong, Xinhui Liu, Jian Yin

**Affiliations:** ^1^ Department of Orthopedics, The Affiliated Lianyungang Hospital of Xuzhou Medical University (The First People’s Hospital of Lianyungang), Lianyungang, China; ^2^ Department of Geriatrics, Jinling Hospital, Affiliated Hospital of Medical School, Nanjing University, Nanjing, China; ^3^ Department of Orthopedics, The Affiliated Jiangning Hospital with Nanjing Medical University, Nanjing, China

**Keywords:** macrophages, osteoclasts, osteolysis, wear particle, aseptic loosening

## Abstract

Joint replacement surgery is the most effective treatment for end-stage arthritis. Aseptic loosening caused by periprosthetic osteolysis is a common complication after joint replacement. Inflammation induced by wear particles derived from prosthetic biomaterials is a major cause of osteolysis. We emphasize that bone marrow-derived macrophages and their fusion-derived osteoclasts play a key role in this pathological process. Researchers have developed multiple intervention approaches to regulate macrophage/osteoclast activation. Aiming at wear particle-induced periprosthetic aseptic osteolysis, this review separately discusses the molecular mechanism of regulation of ROS formation and inflammatory response through intervention of macrophage/osteoclast RANKL-MAPKs-NF-κB pathway. These molecular mechanisms regulate osteoclast activation in different ways, but they are not isolated from each other. There is also a lot of crosstalk among the different mechanisms. In addition, other bone and joint diseases related to osteoclast activation are also briefly introduced. Therefore, we discuss these new findings in the context of existing work with a view to developing new strategies for wear particle-associated osteolysis based on the regulation of macrophages/osteoclasts.

## 1 Introduction

Total joint replacement, which includes hip, knee, shoulder, and ankle replacements, is one of the most effective surgical procedures used by orthopedic surgeons to treat end-stage joint disease. Census data show that the number of total hip arthroplasty (THA) and primary total knee arthroplasty (TKA) in the U.S. will increase from 498,000 and 1.065 million in 2020 to 1.429 million and 3.416 million in 2040, respectively. The Australian Orthopedic Association survey found that the 19-year survival rates for total THA and TKA were 87.8% and 91%, respectively (1). Although significant improvements have been made in surgical methods and prosthetic design, periprosthetic osteolysis (PPO) and prosthetic loosening (PL) due to various reasons are important reasons for long-term surgical failure. By 2060, revisions of THA and TKA are expected to reach 110,000 and 253,000 cases in the U.S, respectively (2). However, revision surgery takes a physical and emotional toll on patients and increases economic pressure on families, society, and the healthcare system. As the life expectancy of joint replacement patients increases, the service life of artificial joints becomes more and more important, accordingly, the prevention of PPO will become an effective treatment for PL. At present, there is no effective drug for the prevention and treatment of PPO in clinical practice.

Mechanical factors and/or biological responses are the two main causes of AL. According to the presence or absence of microbial infection, PL can be divided into infectious loosening and aseptic loosening. Osteolysis caused by wear particles falls into the latter category. In the following sections, non-sterile osteolysis will be briefly discussed. Biological responses induced by wear particles released by artificial joint components at the bone-implant interface are key factors leading to the progression of PPO and PL (3, 4). The material of the wear particles is metal (titanium), alloy (cobalt chromium molybdenum), bone cement, polyethylene or ceramic (5), and the size varies from submicron to hundreds of microns. Although the material of joint prosthesis has been iterated several times, traditional ultra-high molecular weight polyethylene (UHMWPE) has been the main component of joint prosthesis due to its low cost, good biocompatibility, low coefficient of friction, along with high compressive and impact strength. Millions of joint replacements worldwide still contain UHMWPE (6, 7). There is currently no material that does not generate wear debris, and the challenge of wear debris-related biological responses is ongoing. Studies have shown that wear particles with a diameter of 0.1-2.0μm are the most biologically active triggers for inflammatory responses (8). Periprosthetic membrane (PM), formed at the bone-prosthesis interface after joint replacement, is primarily a dense collagen network formed by fibroblasts, in which immune and nonimmune cells contribute to the long-term homeostasis of periprosthetic tissues through inflammatory responses, angiogenesis, collagen deposition, and fibrous tissue remodeling. After phagocytosis of wear particles, macrophages, osteoblasts, osteoclasts, and fibroblasts in PM release a variety of inflammatory factors, such as tumor necrosis factor (TNF)-α, interleukin (IL)-1β and IL-6, which expand the inflammatory storm. These cells secrete proinflammatory mediators and chemokines, including tumor necrosis factor-α (TNF-α), macrophage colony-stimulating factor (M-CSF), IL-1β, IL-6, IL- 17a, prostaglandin E2 (PGE2), vascular endothelial growth factor (VEGF), and RANKL further promote osteoclast formation, differentiation, and maturation, shifting bone metabolic homeostasis to osteolysis, which ultimately leads to periprosthetic bone resorption and osteolysis (9–11). During this pathological process, upregulation of receptor activator of nuclear factor kappa-B ligand (RANKL) and inhibition of osteoprotegerin (OPG) accelerate osteoclast maturation and bone resorption (12). Macrophages are key cells in specific innate immune responses (13). Osteoclasts formed by the fusion and differentiation of macrophages are known as master bone sculptors and are the only and powerful osteolytic effector cells. Inflammatory effects mediated by macrophages play a pivotal role in the development of PPO and AL. Therefore, it is of great clinical significance to elucidate the molecular mechanism of macrophages in aseptic osteolysis and to explore potential strategies for the treatment and prevention of PPO.

## 2 Macrophages

Tissue-resident macrophages (TRM) are distributed in various organs, such as Kupffer cells in the liver; microglia in the central nervous system; Langerhans cells in the skin; bone marrow macrophages and osteoclasts in the bone and alveolar macrophages, which are sentinels of the immune system (14–18). Macrophages maintain bone tissue homeostasis through osteoimmunology regulation (13). Although macrophages, osteoblasts, osteoclasts, fibroblasts, and dendritic cells are involved in wear particle-induced PPO, monocyte-macrophage immune surveillance, phagocytosis and antigen presentation considered primary and crucial (13, 19, 20). Continuously produced wear particles on the bone-prosthesis interface are phagocytized by bone marrow-derived macrophage (BMDMs), exerting innate immunity (nonspecific immunity), which on the one hand leads to increased expression of cytokines (TNF-α, IL-1, IL-6 and TNFSF15), reactive oxygen species (ROS) and proteases, on the other hand, activates the pro-inflammatory M1 phenotype and osteoclasts (21–24). The result is osteolysis around the prosthesis, the destruction of the bony structure supporting the prosthesis, and eventual loosening of the prosthesis. However, the molecular mechanisms by which macrophages recognize wear particles and subsequently induce an inflammatory response have not been fully elucidated. Current studies have revealed that NF-κB signaling pathway is one of the key pathways (25, 26). Thus, regulating the upstream and downstream molecules of the NF-κB signaling pathway is considered to be an important target for the prevention and treatment of PPO (27).

## 3 Osteoclasts

As the rigid structure responsible for movement, protection and support of vital organs, bones are dynamic organs. Bone homeostasis is maintained by the coordination among osteoblasts, osteocytes and osteoclasts. Osteoblasts derived from the mesenchymal lineage secrete a large amount of bone matrix proteins to form a mineralized network, and the osteoblasts encapsulated in it evolve into terminally differentiated osteocytes. Osteoclasts are also members of the myeloid system, which precisely complete bone resorption by tightly attaching to the bone surface and secreting acids as well as proteases. An actively resorbing osteoclast possesses a unique cytoskeletal organization, the actin ring, that forms a bone resorption lacuna in a sealed area (28). Under normal physiological conditions, osteoclasts are critical for maintaining calcium homeostasis, a bone matrix of proper strength and bone remodeling. In addition, osteoclasts promote endochondral bone growth by removing calcified cartilage beneath the growth plate. Stimulatory signals, however, lead to abnormal activation of osteoclasts, leading to pathological bone loss. Several types of pathological osteolysis have been described below.

Monocyte-macrophage fusion forms multinucleated giant cells, which are precursors of osteoclasts. Macrophage CSF (M-CSF), also known as CSF-1, is a dimeric glycoprotein that plays an important role in the proliferation and division of monocytes. CSF-1 is a key factor in osteoclastogenesis, which can promote the expression of receptor activator of nuclear factor kappa B (RANK), a TNF receptor family member, in osteoclast precursors (29, 30). Intriguingly, RANKL is responsible for the fusion of osteoclast precursor cells and differentiation to the osteoclast lineage. RANKL, a membrane-bound and soluble TNF family member, can be produced by a variety of cells, including adipocytes, B and T lymphocytes, chondrocytes, as well as vascular endothelial cells (31). The combination of RANKL and RANK activates NF-κB and mitogen activated protein kinase (MAPK) pathways through TNF receptor-associated factor 6 (TRAF 6), which activate activator protein 1 (AP-1), a key molecular for initiation of nuclear factor of activated T cells c1 (NFATc1) transcription (32, 33). NFATc1 is a master transcription factor in osteoclastogenesis (34). However, increased transcription of NFATc1 is not sufficient to effectively promote osteoclast formation. Downregulation of inhibitory signals including IRF-8, BCL-6 and MAFB is also critical (35). These negative regulators are repressed by BLIMP1. Thus, increased expression of BLIMP1 can promote osteoclast activation (36). In addition to NFATc1, other osteoclast-related genes, such as *Dcstamp*, *VA TPase d2*, *Acp 5*, *MMP9* and *c-Fos*, are activated upon RANKL/RANK binding to accelerate osteolysis of osteoclasts (37, 38). Osteoprotegerin (OPG), a soluble extracellular protein, is a soluble decoy receptor that can bind to RANKL to inhibit all its known functions (39, 40). The imbalance of RANKL/OPG is an important mechanism leading to bone loss. Therapies that antagonize RANKL, such as the anti-RANKL antibody denosumab, are an effective way to treat osteolytic diseases (41). Wear particles around the prosthesis stimulate macrophage fusion and differentiation into osteoclasts. RANKL produced by inflammatory cells competes with OPG produced by osteoblasts to bind to RANK on the surface of monocyte-macrophages, thereby initiating osteolysis (42, 43).

**Figure 1:**
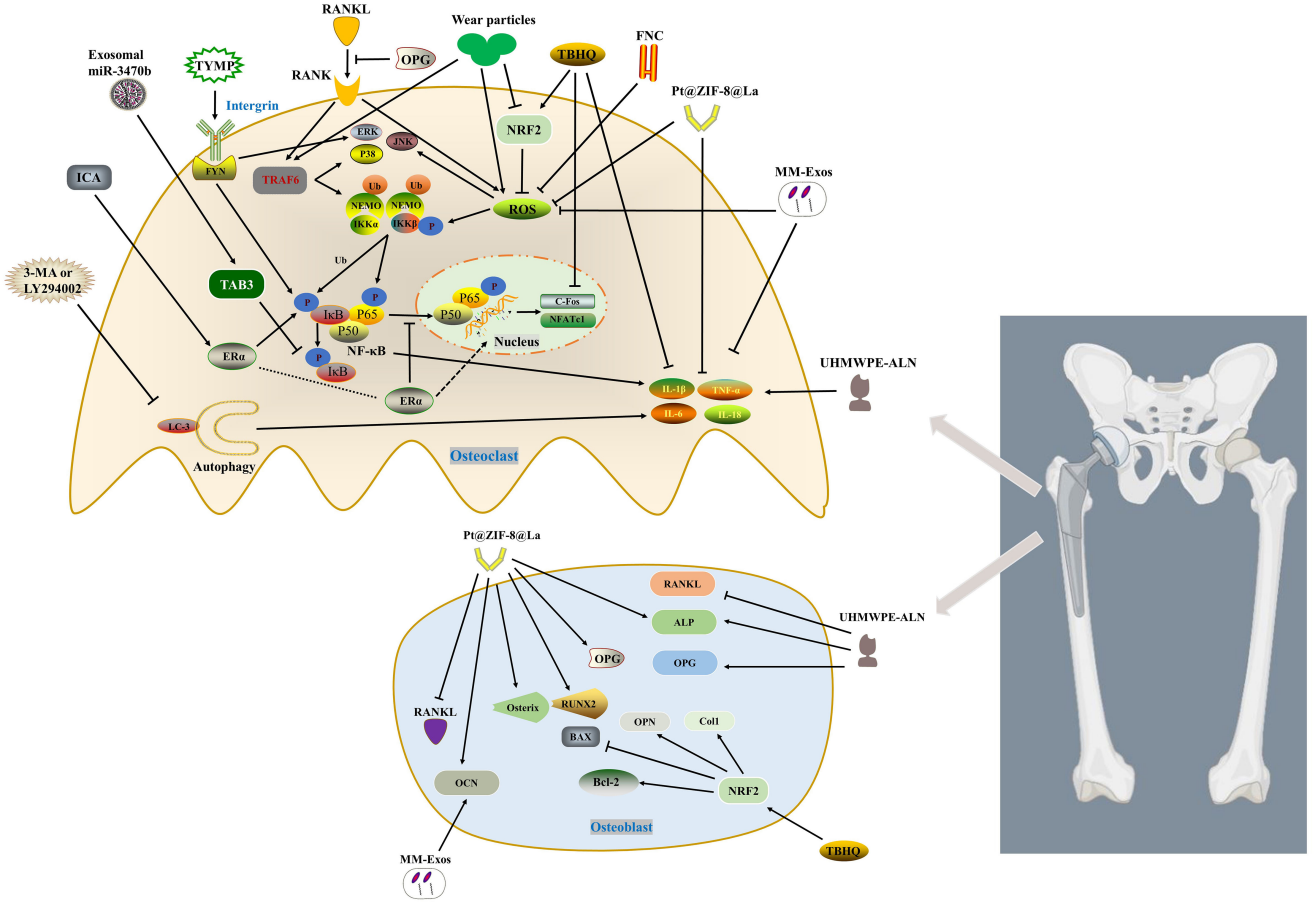
Inhibits osteoclast activation by regulating RANKL/MAPKs (ERK, JNK and p38)/NF-κB, ROS and inflammation pathway. Macrophage-derived exosomes enriched in miR-3470b inhibit NF-κB pathway by targeting TAB3. TYMP promotes osteoclast differentiation by activating integrin-FYN, thereby initiating MAPK and NF-κB signaling pathways. ICA promotes the expression of p-IKKβ, p-p65 and p-IκBα through ERα, and inhibits TNF-α and IL-6. ERα translocates from the cytoplasm to the nucleus thereby inhibiting the translocation of P65 to the nucleus. FNC inhibits p65, p38 and JNK phosphorylation by reducing the expression of ROS. MM-Exos reduce the production of ROS and inflammatory factors (IL-6 and TNF-α) as well as promote the expression of OCN. TBHQ activates NRF2, which on the one hand inhibits osteoclast differentiation by reducing the accumulation of ROS and inflammatory factors (TNF-α and IL-1β), on the other hand promotes osteoblast Col1, OPN, Bcl-2, along with BAX inhibition. Pt@ZIF-8@La scavenges ROS to impede p-Akt and reduce the phosphorylation and degradation of IκBα, while downregulating the levels of IL-1β and TNF-α. Identically, it hinders the expression of RANKL and increases the levels of OPG and osteogenic factors. ALN from UHMWPE-ALN not only reduces the expression of TNF-α, IL-6 and IL-1β, but also promotes the expression of osteoblast ALP and OPG, and inhibits RANKL. 3-MA or LY294002 reduces the activation of inflammatory factors (TNF-α, IL-1β and IL-6) and osteoclasts by inhibiting autophagy.

The current research on the mechanism of macrophages/osteoclasts in wear particle-induced aseptic osteolysis mainly focuses on three types of mechanisms, namely RANKL/MAPKs/NF-κB pathway, reactive oxygen species/antioxidation and inflammation. These three types of mechanisms will be discussed separately below, nevertheless, they are not independent molecular mechanisms (**Figure 1**).

## 4 Inhibiting RANKL/MAPKs (ERK, JNK and p38)/NF-κB pathway

NF-κb is a ubiquitous transcription factor that is directly involved in cytoplasmic/nuclear signal transduction and has direct regulatory effects on osteoclast activation, inflammation and PPO (44–46). Osteoclast differentiation is essentially controlled by the RANKL, mainly via NF-κB and NFATc1.

Exosomes and non-coding RNA (non-coding RNA, ncRNA) have great potential in promoting bone repair, remodeling and regulating bone metabolism (47, 48). Exosomes are bilayered lipid vesicles with a diameter of 50-150 nm produced by the shedding of intracellular compartments or plasma membranes, and carry out signal transduction between cells. For example, miR-214-3p-enriched exosomes derived from osteoclasts inhibit osteoblast bone formation after being taken up by osteoblasts (47). Due to the multiple advantages of exosomes, including lower immunogenicity, wide range of sources, and easy uptake, exosomes have also been extensively studied in the field of osteolysis. Transforming growth factor-β (TGF-β)-activated kinase 1 (TAK1)-binding protein (TAB) 1, TAB2, and TAB3 are all necessary for NF-κB activation. TAB1 binds to the N-terminus of TAK1, whereas TAB2 or TAB3 binds to the C-terminus of TAK1. TAB3 can activate NF-κB through a TRAF6-TAK1-dependent pathway (49, 50). B Pan et al. (51) unmasked that macrophage-derived Exos enriched for miR-3470b rescued Ti particle-induced osteoclast differentiation by targeting TAB3 to inhibit NF-κB pathway.

Bulk RNA sequencing (RNA-seq) has become a powerful weapon for current researchers by identifying novel genes and elucidating their involvement in related signaling networks in orthopedic diseases (52). Since stimulated macrophages expressed gene signatures of rheumatoid arthritis, G Matsumae et al. (53) used RNA-seq analysis to identify 12 target molecules that were highly expressed in rheumatoid arthritis. The results of osteoclast differentiation experiments indicated that thymidine phosphorylase (TYMP) had the highest potential to induce osteoclast differentiation. Strikingly, the increased expression of TYMP in periprosthetic tissue and serum of patients with aseptic loosening further confirmed its potential role as an osteoclastic factor. In a model of cranial osteolysis, TYMP induced bone resorption lesions comparable to RANKL. TYMP induces osteoclasts through the integrin-FYN signaling pathway, thereby activating MAPK and NF-κB signaling pathways. Oral administration of the FYN kinase inhibitor saracatinib could significantly alleviate osteolysis induced by UHMWPE particles.

Icariin (ICA), a flavonoid compound with estrogen-like properties, can antagonize RANKL and estrogen deficiency-induced osteolysis (54–56). Previous studies demonstrated that ICA not only promoted MC3T3-E1 differentiation and mineralization through estrogen receptor-mediated ERK and JNK, but also prevented bone loss caused by estrogen deficiency via activating STAT3 (55, 57, 58). ICA delayed the progression of osteolysis and decreased the expression of TNF-α, IL-1β, and IL-6 in a wear particle-induced calvarial osteolysis model (59). In order to better simulate the actual clinical situation, G Fu et al. (60) extracted and prepared lipopolysaccharide-free wear particles from discarded CoCrMo femoral head implants in clinical revision patients by manufacturing high-vacuum three-electrode direct current. The wear particles and ICA were used *in vivo* and *in vitro* experiments, and the results suggested that ICA could significantly reduce the protein expression levels of p-IKKβ, p-p65 and p-IκBα, and promote the expression of phospho-ERα Ser118 and phospho-ERα Ser167 proteins. In addition, ERα translocated from the cytoplasm to the nucleus, thereby inhibiting the translocation of P65 to the nucleus. ICA also reversed the expression of TNF-α and IL-6 mediated by wear particles through the NF-κB signaling pathway (60). Recent findings suggested that piperlongumine inhibited osteoclast formation and bone resorption by repressing the activation of MAPKs (ERK, JNK, p38) and NF-κB induced by RANKL as well as downregulating the expression of NFATc1 protein (61). Herein, inhibition of the RANKL/MAPKs (ERK, JNK, and p38)/NF-κB pathway in macrophages/osteoclasts is considered a promising approach for the prevention and treatment of PPO.

## 5 Anti-oxidation and hindering ROS formation

It is well established that the proper intensity of redox reactions is critical to the homeostasis of various organs, including the skeletal system (62, 63). Under normal physiological conditions, an appropriate level of ROS acts as a second messenger of cell signal transduction to facilitate the maturation of osteoclasts (64). However, oxidative stress leads to enhanced osteoclast activation (64, 65). Wear particles stimulate the local immune-inflammatory response, activate and recruit macrophages to produce excess ROS containing unpaired electrons, such as hydroxyl radicals, hydrogen peroxide, superoxide anion, hydrogen peroxide superoxide anion, singlet oxygen and hydroxyl radicals (66). During inflammation, ROS maintain M1 macrophage polarization (67). Studies have identified that ROS enhances osteoclast differentiation through NF-κB and MAPK pathways (64). RANKL induces increased ROS production in BMDMs through the TRAF6/Nox1 signaling pathway. Accumulated ROS on the one hand oxidizes tyrosine phosphatases, thereby inducing MAPK phosphatase (MKPs) inhibition and activation of MAPKs (68). On the other hand, it promotes the homodimerization of LC8, leads to the dissociation of LC8 and IκBα, and then increases the phosphorylation and degradation of IκBα, consequently, promoting the nuclear translocation of NF-κB dimer (69, 70). Accordingly, reducing and scavenging excess ROS to inhibit osteoclast activation is a realistic strategy to alleviate wear particle-induced aseptic osteolysis.

ROS scavenging depends on antioxidant enzymes such as superoxide dismutase (SOD), peroxidase (POD) and catalase (CAT) (71, 72). Nuclear factor-erythroid 2 related factor 2 (Nrf2), a redox-related transcription factor, promotes the gene expression of multiple antioxidant enzymes through translocation to the nucleus (73–75). Previous study has suggested that Nrf2 deficiency in BMDMs leads to ROS accumulation and exacerbated osteolysis (76). Targeting Nrf2 has been unmasked to effectively inhibit osteoclast differentiation and bone resorption (77–79). Enhancing endogenous antioxidants to reduce ROS and the downstream molecules formation may effectively counteract the adverse effects of wear particles.

Oxidative stress inhibits the osteogenic effect of osteoblasts and promotes the formation of osteoclasts (63). Nrf2, a key regulator of oxidative stress, not only regulates the transcription of antioxidant enzymes, including glutathione reductase, SOD, glutathione oxidase and CAT, etc., but also inhibits the inflammatory response of macrophages by blocking the transcription of pro-inflammatory cytokines (80–83). J Dong et al. (84) found that metal wear particles significantly inhibited the expression of NRF2 during the process of calvarial osteolysis. In mice with *NRF2* gene (Nfe2l2) knockout, the expressions of NFATc1 and cathepsin K (CTSK) in osteoclasts were enhanced after Ti particle stimulation, simultaneously, collagen 1 (Col1), Osteopontin (OPN) and apoptotic proteins were increased in osteoblasts. Compared with the more severe osteolysis caused by Nfe2l2 knockout, the NRF2 agonist tert-butylhydroquinone (TBHQ) could block ROS accumulation and effectively correct the effects of NRF2 deficiency on osteoclasts and osteoblasts, thereby reversing metal particle-induced inflammation and oxidative osteolysis.

Although Exos have made important progress in skeletal diseases and spinal cord injuries (85, 86), which show great promise in the field of tissue damage repair and regeneration, their clinical applications are still limited. Exosomes lack tissue and organ targeting and are easily phagocytized by immune cells such as macrophages. Encapsulating drugs through the macrophage membrane can avoid being cleared by the autoimmune system, which has a higher target delivery efficiency for inflammatory diseases (87–89). Some scholars have found that macrophage membrane-encapsulated urine-derived stem cell-derived exosomes (MM-Exos) can be targeted and delivered to the osteolytic zone, which can not only promote the osteogenic differentiation of bone marrow-mesenchymal stem cells (BMSCs), but also inhibit ROS, RANKL, IL-6 and TNF-α production, thereby attenuating UHMWPE-induced osteolysis (90). By providing immune camouflage to Exos to enhance delivery efficiency, it provides a new drug delivery system for the treatment of inflammatory diseases, including osteolysis.

K Sun et al. (91) developed a new antioxidant, few-layered Nb_2_C (FNC), based on ternary metal carbide/nitride, which reduced cytokine production and inhibited osteoclastogenesis by adsorbing ROS. FNC was able to inhibit the phosphorylation of NF-κB p65, p38 and JNK, but not ERK(1/2), after LPS or RANKL stimulation. Although the conclusion may support that FNC regulated inflammation (IL-1β and IL-6 involved) or bone resorption through NF-kB and MAPKs signaling pathways, the authors did not interfere with this target and further confirm it.

A bimetallic organic framework (Pt@ZIF-8@La) loaded with platinum (Pt) nanozyme with ROS scavenging and anti-inflammatory capabilities as well as osteogenic active element lanthanum (La) was constructed for Ti-induced calvarial osteolysis model. Pt@ZIF-8@La exhibited strong ROS scavenging ability both *in vivo* and *in vitro*. On the one hand, Pt@ZIF-8@La inhibited the p-Akt of RAW 264.7 cell line to rescue the phosphorylation and degradation of IκBα, and decreased the expression levels of NO, IL-1β, and TNF-α. On the other hand, while increasing the expression of OPG and the ratio of OPG/RANKL in MC3T3-E1 cells, it also promoted the expression of osteogenesis-related genes ALP, RUNX2, Osterix and OCN (92). It seems to be an effective way to promote the osteogenic effect of osteoblasts while inhibiting the oxidative stress and inflammatory factors mediated by osteoclast ROS.

## 6 Inhibiting inflammation

Wear particle-induced inflammation is the central process in osteolysis and aseptic loosening. Toll-like receptors (TLRs) not only recognize exogenous pathogen-associated molecular patterns (PAMPs), but also detect endogenous products associated with inflammation, such as heat shock proteins, high mobility base box (HMGB) 1, fibronectin Protein and Hyaluronic Acid. Adenosine triphosphate (ATP) and ROS can further activate NLRP3, a member of the nucleotide-binding oligomerization domain (NOD)-like receptor (NLRs) family, which intensifies and perpetuates the process of inflammation (93–95).

Alendronate sodium hydrate (ALN), an inhibitor of farnesyl diphosphate synthase, is a commonly used anti-osteoporosis drug in clinical (96). Y Liu et al (97). loaded alendronate sodium on critical size UHMWPE (UHMWPE-ALN), and used alginate-coated beads as a cell reactor to co-culture cells and UHMWPE-ALN wear particles. On the one hand, the release from UHMWPE-ALN particles inhibited the expression of TNF-α, IL-6, IL-1β inflammatory factors and the proliferation of RAW264.7, on the other hand, it promoted the osteogenesis of osteoblasts and the level of OPG and down-regulated RANKL gene expression. Unfortunately, the author did not conduct the necessary confirmation through animal experiments. Although this drug-loading method provides a new idea for the treatment of PPO, the stability of drug release and the negative effects of long-term drug effects are issues that need to be considered in clinical applications.

Autophagy, as a highly conserved process of self-degradation and energy dynamic cycle unique to eukaryotic cells, plays an important role in PPO (98). However, autophagy is a double-edged sword, which may bring different results at different stages of PPO development and in different cells (98). W Chen et al. (99) found that Ti particle-induced osteoclastogenesis and decreased expression of TNF-α, IL-1β and IL-6 could be alleviated by inhibiting autophagy (3-MA, LY294002 or knocking out Atg5 gene). Relevant literatures are summarized in **Table 1**.

**Table 1:** Experimental study of macrophages/osteoclasts in attenuating wear particle-induced aseptic osteolysis.

Intervention target/agents	Key Molecules or Pathways	Animal model	Cell model	Molecular mechanism	Ref
Macrophages-derived exosomes	TAB3, NF-κB signaling	Calvarial osteolysis induced by Ti particles	Macrophages stimulated by Ti particles co-cultured with osteoclasts and osteoblasts respectively	Exosomal miR-3470b inhibits osteoclast differentiation by targeting TAB3/NF-κB.	51
TYMP	FYN, c-Myc, P65, RELB, MEK1/2, ERK1/2, NF-κB	Calvarial osteolysis induced by UHMWPE or TYMP	Macrophages stimulated by UHMWPE	TYMP promotes osteoclast differentiation by activating integrin-FYN, thereby initiating MAPKs and NF-κB signaling pathways.	53
Icariin	NF-κB, Erα, TNF-α, IL-6	Calvarial osteolysis induced by CoCrMo	BMDMs stimulated by CoCrMo	ICA alleviates osteolysis and inflammatory factors through ERα-mediated NF-κB signaling pathway.	60
FNC	ROS, p65, p38, JNK	Calvarial osteolysis induced by UHMWPE	BMDMs stimulated by M-CSF and RANKL	FNC inhibits osteoclast activation and osteolysis by reducing ROS generation. NF-κB and MAPKs signaling pathways may be involved.	90
MM-Exos	ROS, RANKL, IL- 6, TNF- α	Calvarial osteolysis induced by UHMWPE	RAW264.7 stimulated by UHMWPE and RANKL	MM-Exos can be targeted and enhance the effect of exosomes. MM-Exos not only inhibits osteoclastogenesis by reducing ROS, IL-6 and TNF-α, but also promotes the expression of osteogenic OCN.	89
NRF2	ROS, IL-1β, TNF-α, Bcl-2, BAX, OPN, Col1	Calvarial osteolysis induced by Ti particles or Co-particles	Osteoclast (from BMDMs stimulated by M-CSF and RANKL) and osteoblasts stimulated by Ti particles	Deletion of NRF2 promotes osteoclast differentiation and inhibits osteogenesis of osteoblasts. TBHQ enhances the anti-osteolytic effect of NRF2 by blocking ROS accumulation.	83
Pt@ZIF-8@La	ROS, IL-1β, TNF-α, OCN RUNX2, OPG, Osterix,	Calvarial osteolysis induced by Ti particles	RAW264.7 and MC3T3-E1 stimulated by Ti particles	Pt@ZIF-8@La scavenges ROS to relieve inflammation of RAW 264.7 and promote the ratio of OPG/RANKL in MC3T3-E1 cells and osteogenic effect.	91
UHMWPE-ALN	TNF-α, IL-6, IL-1β	*Not implemented*	RAW264.7 stimulated by UHMWPE	ALN released from UHMWPE-ALN wear particles inhibits the expression of TNF-α, IL-6, and IL-1β and promotes RAW264.7 apoptosis, while promoting osteoblast OPG and down-regulating RANKL.	96
3-MA, LY294002, Atg5 gene knockout	TNF-α, IL-1β, IL-6	Calvarial osteolysis induced by Ti particles	RAW264.7 stimulated by RANKL	Inhibition of autophagy alleviates osteoclast activation.	98

ALP, alkaline phosphatase; BMDMs, bone marrow derived macrophages; Col1, collagen 1 ; Co-particles, cobalt-chromium-molybdenum alloy particles; Erα, estrogen receptor α ; FNC, few-layered Nb_2_C, a new type of antioxidant; FYN, tyrosine kinase; ICA, icariin; MM-Exos, macrophage membrane encapsulated urine-derived stem cell-derived exosomes; NRF2, nuclear factor erythroid derived 2-related factor 2; OCN, osteocalcin; OPG, osteoprotegerin; Pt@ZIF-8@La, platinum@zinc imidazolium zeolite framework-8@lanthanum; RANKL, receptor activator of nuclear factor kappa-B ligand ; ROS, reactive oxygen species; TAB3, transforming growth factor-β (TGF-β)-activated kinase 1 (TAK1)-binding protein 3; TBHQ, tert-butylhydroquinone, an NRF2 agonist ; Ti, titanium; TYMP, thymidine phosphorylase; UHMWPE, ultra-high molecular weight polyethylene ; UHMWPE-ALN, UHMWPE loaded with alendronate sodium.

## 7 Other forms of osteolysis mediated by macrophages/osteoclasts

In addition to wear particle-induced aseptic osteolysis, abnormal activation of osteoclasts is also closely related to a variety of other bone diseases, including osteoporosis, primary and metastatic bone tumors, rheumatoid arthritis, and bone infection. The following will briefly introduce these types of osteolysis.

### 7.1 Osteoporosis

Osteoporosis is a systemic disease characterized by bone loss, decreased bone density, and microarchitectural deterioration of bone tissue, leading to an increased risk of fracture (32, 100). It is estimated that in the United States, more than 25 percent of women over the age of 65 have osteoporosis, compared with 5 percent of men (101). In China, the prevalence of osteoporosis in women over 50 years old is 29.13% (102). The pathogenesis is usually due to a combination of abnormal osteoclast activation and osteoblast dysfunction. Estrogen has an antagonistic effect on osteoclasts. Ovary secretion of estrogen decreases in postmenopausal women, leading to hyperactivation of osteoclasts. Therefore, selective estrogen receptor modulators (SERMs), such as raloxifene and strontium ranelate, can exert estrogen receptor-mediated bone protection (103, 104). However, studies have also found that exogenous estrogen intake is a potential cause of breast cancer and other tumors (105). The most important treatment for osteoporosis is to block the occurrence and cell function of osteoclasts. In addition to estrogens, bisphosphonates and RANKL inhibitors are also being developed. The application of these drugs will inevitably bring certain side effects. For example, bisphosphonates can cause hypocalcemia, osteonecrosis of the jaw, and atrial fibrillation (106, 107). Denosumab, a human anti-RANKL antibody, potently blocks the interaction between RANK and its ligands to inhibit bone resorption, but long-term use can lead to infection, rash, and atypical femoral fractures (108, 109). Due to the long half-life of Denosumab and the need for parenteral administration, Morikawa N et al. (110) developed a new oral small molecule RANKL signal transduction inhibitor, AS2690168, which can simultaneously reduce RANKL-induced NFATc1 mRNA expression in RAW264 cells, inhibit parathyroid hormone-stimulated calcium release from the skull of mice, and alleviate the decline in femoral BMD in ovariectomized rats.

Some scholars have also found that multiple histone deacetylases and histone acetyltransferases are expressed during osteoclast differentiation, which are considered potential targets for the treatment of osteoporosis (111, 112). In recent years, a series of new drugs have been applied to inhibit osteoclast differentiation and anti-osteoporosis in LPS-induced mouse calvarial bone loss model and ovariectomy (OVX)-induced osteoporosis model. For example, Saikosaponin D, the active extract of Bupleurum bupleuri, decreases the expression of genes related to osteoclast differentiation and function, including VA TPase d2, Dcstamp, acp5 and c-Fos (113). Another small molecule compound extracted from natural plants, methyl 3,4-dihydroxybenzoate (MDHB), can promote Nrf2 expression by reducing ubiquitination-mediated proteasomal degradation of Nrf2 and reducing ROS levels, thereby inhibiting RANKL-induced activation of MAPK and NF-κB pathways (114). Tetrandrine inhibits osteoclast bone resorption by enhancing the ubiquitination degradation of TNF-related apoptosis-inducing ligand (TRAIL) and inhibiting the phosphorylation of p38, p65, JNK, IKBα and IKKα/β (115). Ceritinib is used to treat anaplastic lymphoma kinase (ALK)-rearranged non-small cell lung cancer, which inhibits RANKL-induced phosphorylation of Akt and p65 in osteoclast and improves trabecular bone loss in OVX-mice (116). Collectively, these newly developed drugs inhibit osteoclast mainly through ROS, MAPK, and NF-κB pathways.

### 7.2 Bone tumor

Osteolytic bone metastases are common in solid tumors, including lung and breast cancer, and are one of the direct causes of death (117). About 30-40% of patients with non-small cell lung cancer develop bone metastases, and even more than 90% in prostate cancer (118, 119). PTHrP, IL-11, IL-6, and TNF-α secreted by tumor cells induce hyperactivation of osteoclasts, giving preference to osteolysis over osteogenesis (120). Although prostate cancer bone metastasis ultimately manifests as an osteogenic effect, accumulating evidence suggests that accelerated bone resorption induced by osteoclasts is the key to tumor bone metastasis (121–123). Drugs targeting osteoclast inhibition, such as bisphosphonates and Denosumab, have been tried for the treatment of osteolytic bone tumors, while clinical benefits are limited by high cost, side effects and little long-term benefit (124, 125).

Multiple myeloma (MM) is characterized by malignant clonal expansion of plasma cells, and osteolytic destruction is a classic hallmark. Osteolytic lesions occurred in more than 80% of patients, accompanied by hypercalcemia, pathological fractures, bone pain and spinal cord compression, which seriously threatened the life of patients (126). Increased osteoclast-mediated bone resorption is accompanied by decreased osteoblast-mediated bone formation in patients with multiple myeloma (127), the molecular mechanism of which has not yet been elucidated.

Cystatin M/E (CST6), a cysteine ​​protease inhibitor, belongs to the type 2 cystatin family and inhibit Cathepsin B (CTSB), Cathespin L (CTSL) and Legumain (LGMN) proteases (128). CST6 is considered a tumor suppressor due to its epigenetic silencing in tumors along with inhibition of cancer cell proliferation and metastasis (129). Study has implied that MM cells secrete CST6 to block RANKL-induced osteoclast maturation by inhibiting cathepsin-mediated cleavage of NF-κB/p100 and TRAF3, thereby alleviating bone loss in MM patients (130). Furthermore, breast cancer cell-derived CST6 enters osteoclasts through endocytosis and upregulates the hydrolysis substrate of CTSB, SPHK1, by inhibiting CTSB. Sphingosine kinase 1 (SPHK1) inhibits RANKL-induced p38 activation, thereby hindering osteoclast maturation and breast cancer bone metastasis (129). More and more researches have revealed that the expression of CST6 can prevent bone metastasis of tumors (129, 131). Accordingly, CST6 is considered to be a new type of anti-resorptive agent for the treatment of osteoclast-mediated osteolysis.

Increasing evidences imply that cargoes of extracellular vesicle (EV), such as microRNAs, can serve as “messengers” for communication between tumor cells and osteoclasts. Prostate cancer cell-derived miR-378a-3p-enriched EVs are taken up by macrophages, and miR-378a-3p promotes nuclear translocation of Nfatc1 by inhibiting Dyrk1a, thereby accelerating osteolysis (132). Sclerostin, a small glycoprotein encoded by the *Sost* gene, is secreted mainly by osteocytes and is essential for osteoblast development (133). Sclerostin also plays an important regulatory role in bone formation and bone resorption (134). Intriguingly, sclerostin and RANKL secreted by osteocytes are significantly elevated in circulating serum of multiple myeloma patients (135). The study has unmasked that 2-deoxyD-ribose derived from myeloma cells promoted the expression of major histocompatibility complex class II transactivator (CIITA) in osteocytes through the STAT1/IRF1 signaling pathway. CIITA induces hyperactivation of osteoclasts by increasing the secretion of osteolytic cytokines by osteocytes through acetylation of histone 3 lysine 14 in the promoters of TNFSF11 (encoding RANKL) and SOST (encoding sclerostin) (136). Inhibition of sclerostin expression significantly reduces osteolytic bone lesions in a mouse model of myeloma (134). Consequently, therapeutic targeting of anti-sclerostin has also been attempted for the treatment of osteoclast-associated osteolytic diseases. However, the mechanism by which osteocytes secrete osteolytic cytokines under the stimulation of tumor cells remains to be elucidated.

### 7.3 Rheumatoid arthritis

Rheumatoid arthritis (RA) is an autoimmune disease common in women, with an incidence of about 1% (137). Long-term chronic inflammation leads to osteoclast activation and degeneration of cartilage and bone tissue. During the pathological process of the disease, the release of RANKL by activated lymphocytes induces osteoclast activation and the release of TNF-α and IL-6 (138). Another autoimmune disease, psoriatic arthritis (PsA), has a similar cellular mechanism (139). Study has exhibited that CD83 can reduce the expression levels of RANKL, OC-Stamp, MMP9, IL-1b and IL-6 by inducing the downstream of the signaling cascade to bind to the toll-like receptor 4/(TLR4/MD2) receptor complex, thereby inhibiting osteoclast formation and preventing arthritic bone erosion (140).

### 7.4 Bacterial infection of bone

Just as its name implies, aseptic osteolysis is defined first by absence of evidence of clinical or microbial infection and second by compliance with clinical symptoms and radiographic evidence (141, 142). Despite significant advances in medicine to combat infection, bone infection remains a formidable threat in orthopedic surgery. In the United States, there are about 10,000-20,000 cases of joint replacement infection and 30%-42% of fracture-related infections each year, and the most important pathogen is Staphylococcus aureus (143, 144). The clinical outcome of infection is mostly surgical failure and revision surgery with a high recurrence rate.

The molecular mechanism of bone infection-mediated osteolysis remains to be elucidated. Existing evidence shows that macrophages are the first line of defense against pathogens, while neutrophils are the main executors of the innate immune response to bacteria. When anti-inflammatory M2 macrophages are activated by pattern recognition receptors (PRRs), PRRs recognize bacterial pathogen-associated molecular patterns (PAMPs), leading to macrophage M1 polarization (145, 146). Macrophages secrete chemokines TNFα, IL-1β, and CXCL1 to recruit and activate neutrophils (147–149). RANKL released by neutrophils can promote osteoclastogenesis (150). Simultaneously, macrophages that phagocytize wear particles increase the release of pro-inflammatory factors, accelerating osteoclast activation and osteolysis.

The essential role of osteocytes in bacterially induced osteolysis depends on the regulation of RANKL release. PAMPs increase the release of RANKL by activating the myeloid differentiation primary response 88 (MYD88) pathway in osteocytes. Inhibition of MYD88 blocks calvarial osteolysis induced by PAMPs and resist alveolar bone resorption induced by oral Porphyromonas gingivalis (Pg) infection. Mechanistically, MYD88 promotes phosphorylation of CREB and STAT3, thereby increasing RANKL release from osteocytes (151). As a pleiotropic cytokine, the role of IL-27 in bacterial infection remains controversial. Exogenous IL-27 administration for Staphylococcus aureus-infected osteomyelitis not only induces accumulation of pro-inflammatory IL-17-producing RORγt+ neutrophils, leading to reduced abscess formation, but also inhibits RANKL-induced osteoclast activation to relieve osteomyelitis osteolysis (152). It has also been confirmed in multiple studies that IL-27 can directly inhibit rank-induced osteoclastogenesis and reduce bone loss (153–158). Macrophages are the host cells of M. tuberculosis infection and the innate immune cells of the host responsible for killing and clearing M. tuberculosis (159). STAT1 and CXCL10 are involved in M1-macrophage polarization and contribute to osteolysis and bone remodeling during extrapulmonary tuberculosis infection (160). In addition, osteopetrosis, a congenital generalized abnormal development of bone structure, is caused by defects in the production of osteoclast factors (161). There is currently no effective treatment for osteopetrosis, although bone marrow transplants and cord blood transfusions hold promise. Notably, the regulation of osteoclasts may provide new therapeutic strategies.

## 8 Disscussion

Total joint arthroplasty (TJA) is the most appropriate way to treat end-stage arthritis, although surgical methods and implanted materials are constantly evolving, aseptic PPO and PL, mainly mediated by wear particles, are the most common causes of TJA failure reason (162). BMDMs, immune cells of the nonspecific innate immune response, and osteoclasts differentiated and fused by BMDMs play a crucial role in periprosthetic bone homeostasis. Phagocytosis of wear particles by macrophages results in the release of proinflammatory cytokines, growth factors, and chemokines. The inflammatory cascade stimulates the maturation of osteoclasts and, meanwhile, inhibits the osteoblast lineage (13). Therefore, the regulation of macrophage and osteoclast function is a potential strategy to optimize the biological and clinical outcomes of joint prosthesis implantation surgery. To date, there are no approved pharmacological interventions that effectively prevent particle-associated periprosthetic osteolysis. Although some anti-osteoclast drugs, such as bisphosphonates, estrogens, and RANKL inhibitors, have been developed, their therapeutic effects are controversial (163, 164). For example, bisphosphonates are used in anti-osteoporotic therapy, while macrophages exposed to zoledronic acid polarize to an M1 pro-inflammatory phenotype, for this reason, the use of bisphosphonates to treat wear particle-associated osteolysis is not theoretically supported (165, 166). Currently, new ways to modulate macrophage/osteoclast activity to replace existing therapies are urgently needed.

The current research on the signaling pathways of macrophages and osteoclasts mainly focuses on the formation of ROS and inflammatory factors mediated by the RANKL/NF-κB/MAPKs pathway. This is a classic signaling pathway that crosstalks with various regulatory cell deaths, such as pyroptosis, autophagy, pyroptosis, and ferroptosis. Many of these unknown mechanisms remain to be elucidated may provide valuable strategies for mitigating PPO. Exosomes can not only stimulate stem cell-like regenerative effects in damaged tissues, but also avoid the risks and drawbacks associated with stem cell transplantation therapy (167, 168). Some stem cell-derived exosomes can promote osteogenic differentiation and inhibit the production of inflammatory factors and osteoclast activation, which has great potential in preventing PPO. Undoubtedly, the recognition and subsequent clearance of exosomes by immune cells in the body is the biggest challenge for the application of exosomes *in vivo*. Recently, research on biomimetic drug delivery systems has attracted more and more attention. Macrophage membrane-coated nanoparticles have high targeted delivery efficiency and show good therapeutic effects on various inflammatory diseases, including wear particle-induced periprosthetic inflammation (88, 169). Currently, there are many types of nanoparticles or drugs coated in macrophage membranes, for example, lactose-acid nanoparticles (170), liposomes (169), poly(lactic-co-glycolic acid) (88), nano-gemcitabine (171), magnetic photothermal nanocomplexes (172), poly lactic-co-glycolic acid nanoparticle vaccine carrying PilY1 Ep (173) and mesoporous silica nanoparticles (174), etc. Macrophage membrane-coated drugs can avoid being recognized and cleared by the immune system, thereby enhancing the stability of drug release (175). Notably, protein molecules on cell membranes may elicit an immune response. These potential safety issues need to be addressed before their successful application in clinical practice.

The constant renewal of materials, such as highly polished cobalt chromium alloy and zirconia ceramics, to reduce the generation of wear debris has achieved a certain effect. Although the wear intensity is relatively reduced, osteolytic wear particles are still unavoidable (176). The development of biomaterials to suppress inflammatory responses and osteoclastogenesis through prosthetic drug delivery provides opportunities for the treatment of PPO. UHMWPE loaded with antioxidant vitamin E can significantly reduce the production of osteolytic cytokines TNFα, IL-1β, IL-6 and IL-8 by peripheral blood mononuclear cells, which reduces the generation of wear particles of implants (43). In the coating on the surface of the prosthesis, such as ceramics and hydroxyapatite, adding antibacterial drugs or metal ions can enhance the antibacterial performance (177–179). Adding bone morphogenetic protein-2 (BMP-2) to the coating can significantly promote osteogenesis (180). Similarly, loading prosthetic coatings with drugs that inhibit inflammation and osteoclast activation may benefit from the same therapeutic strategy.

Despite current knowledge, wear particle-induced periprosthetic osteolysis is an aseptic loosening, ie without evidence of clinical or microbial infection. However, a large body of research evidence also supports the role of bacteria in aseptic loosening (181–183). Macrophages, foreign body giant cells, T cells, and B cells were also present in the tissue surrounding the prosthesis during revision surgery with a diagnosis of aseptic loosening. Some scholars believe that wear-related inflammatory reactions, including sterility and suppuration, are interrelated (184–186). The molecular mechanism may involve the impact of wear debris on macrophages and neutrophils impairing the ability of the innate immune system to clear bacteria. In addition, wear particles destroy dendritic cells and T lymphocytes of the adaptive immune response. Although the role of dendritic cells in the mechanisms of implant debris-induced inflammation remains unclear, they are required for targeted responses to infection in periprosthetic joints (187) In conclusion, the combination of antibiotic drugs, anti-inflammatory drugs and antioxidants to modify implanted prosthetic materials is a potential strategy to reduce the risk of PPO (188, 189).

In summary, orthopedic surgeons must accurately implant prostheses during joint replacement and continuously improve surgical skills. Simultaneously, they also need to pay attention to the outstanding problem of PPO, which leads to long-term failure of surgery. Efficient regulation of macrophages and osteoclasts is a promising way to treat wear particle-mediated PPO.

## Author contributions

ZY: Conceptualization, Investigation, Software, Writing – review & editing, Supervision. GG: Investigation, Supervision, Formal Analysis, Validation, Writing – original draft. XL: Investigation, Writing – original draft, Funding acquisition, Methodology, Resources, Visualization, Writing – review & editing. JY: Investigation, Writing – original draft, Writing – review & editing, Conceptualization, Data curation, Software.
