# Supplementary figures and images for: Mechanism of regulating macrophages/osteoclasts in attenuating wear particle-induced aseptic osteolysis
Source: Front Immunol. 2023 Oct 4;14:1274679. doi: 10.3389/fimmu.2023.1274679 (PMC10582964; doi:10.3389/fimmu.2023.1274679)

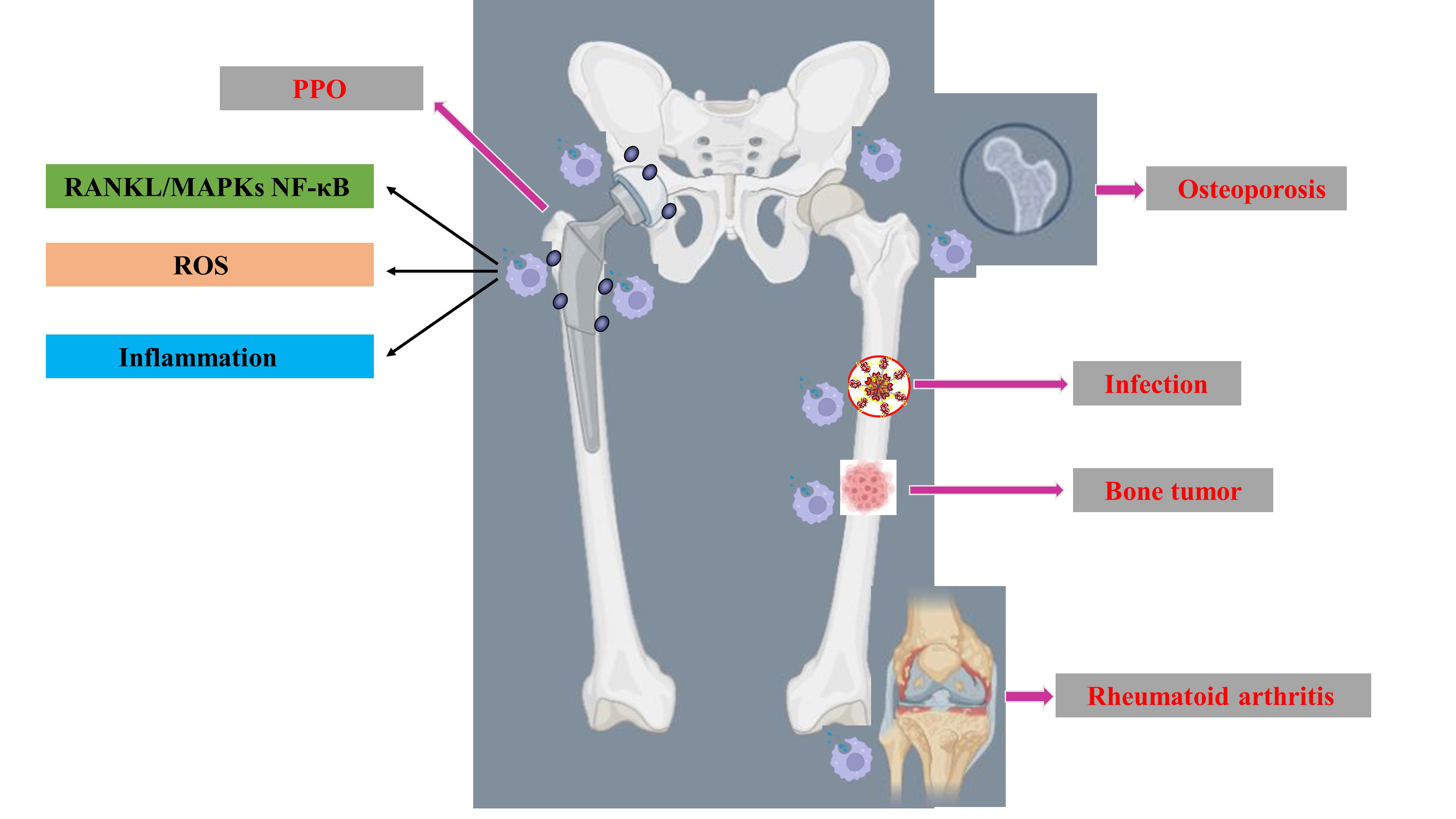

Supplement: Supplementary file 1 [file Image_1.jpeg]
